# Metal economy in host-microbe interactions

**DOI:** 10.3389/fcimb.2014.00190

**Published:** 2015-01-13

**Authors:** Frédéric J. Veyrier, Mathieu F. Cellier

**Affiliations:** ^1^Institut PasteurParis, France; ^2^Institut National de la Recherche Scientifique-Institut Armand FrappierLaval, Canada

**Keywords:** metal, virulence, host, pathogen, transporter, exporter, regulation

This Research Topic presents knowledge on transition metal metabolism in various infections from the dual perspective of offender and defender.

## Host nutritional immunity: depriving or poisoning

To date, the implication of divalent metals have been described in two different immune strategies that aim to fight microbial invaders. One consists in depriving microbes of essential divalent metals whereas the other aims at overloading invaders with toxic concentrations of metal. The contributions in this section present, in different situations, various aspects of this metal economy at the host-microbe interface.

Two papers deal with **metal homeostasis as hosts interact with bacteria**. Diaz-Ochoa et al. (2014) review immunological mechanisms to sequester Fe, Mn, and Zn in the inflamed gut and strategies of commensals and pathogens to evade mucosal defenses and obtain such nutrients. Lisher and Giedroc (2013) detail chemical and structural mechanisms to capture Mn, an antioxidant used by pathogens to adapt to human hosts, and the impact of Fe and Zn on Mn bioavailability during infections.

The most coveted metal, **iron is key to nutritional immunity and microbial virulence**. Using amoeba as model phagocyte, Bozzaro et al. (2013) present the tug of war between a bacterial predator, sequestering intracellular iron to resist invasion, and pathogens which elude such defense mechanisms. On mammalian defense against intracellular bacteria and protozoan parasites, Silva-Gomes et al. (2013) outline divergent approaches: iron-withholding to prevent microbial replication or iron-based oxidative injury to kill invaders.

Host may also target invaders with **toxic doses of Cu and Zn**, normally kept at low concentrations. Neyrolles et al. (2013) present an opinion article on bacterial Zn and Cu poisoning in the context of *Mycobacterium tuberculosis* infection. Chaturvedi and Henderson (2014) summarize the specific properties of copper and its toxic effect on bacteria cells. Arguello et al. (2013) review how bacteria integrate homeostatic mechanisms to avoid Cu toxicity by sensing and regulating ion chelation, chaperoning and membrane transport.

## Microbial adaptation to host defenses: metallo-transporters or exporters

To overcome host resistance to infection, numerous mechanisms have been selected through the course of microbial evolution, in particular transporters that can feed the bacteria even at low metal concentration or, on the contrary, metallo-exporters that can expel metals outside the cell to avoid toxic accumulation. The articles in this section describe the microbial transport arsenal, and its regulation, which play major roles to influence metal economy at the host-microbe interface.

**Bacterial and fungal strategies to acquire Fe** is the subject of four contributions. Liu and Biville (2013) discuss erythrocyte parasitism by *Bartonella*, transmitted by arthropod vectors and relying principally on heme capture and oxidative stress defense to cause persistent infections. Runyen-Janecky (2013) highlights some of the recent findings on heme iron acquisition system and the regulation of their expression in Gram-negative pathogens. Cornelis and Dingemans (2013) recap how Pseudomonas adapts means of iron capture to the type of infection it establishes, acute or chronic. Caza and Kronstad (2013) contrast strategies of virulent bacteria and fungi to subvert host immunity and steal iron from hemoglobin, heme, transferrin and lactoferrin or elemental iron using specialized uptake systems and siderophores.

Five papers deal with **microbial homeostasis of other metals Mn, Ni, and Zn**. Honsa et al. (2013) review the roles of importers and exporters of Mn, Fe, Zn, and Cu in *Streptococcus pneumoniae* gene regulation and tissue-specific pathogenesis. Guilhen et al. (2013) focus on families of exporters and the role of metal efflux in the evolution of *Neisseria meningitidis* virulence and naso-pharyngeal colonization. De Reuse et al. (2013) present the specific nickel needs of the gastric pathogen *Helicobacter pylori* and the homeostasis of nickel in this bacterium. Finally, Zn homeostasis is the subject of two articles. Staats et al. (2013) present the role of Zn in bacteria-host relationship and how this metal represents a very promising target for the development of novel antimicrobial strategies. Cerasi et al. (2013) emphasize the role of Zn in the host-fungi relationships and the impact of Zn bioavailability on the expression of virulence genes.

Lastly, **metallo-regulation of bacterial gene expression** is discussed in relation to virulence. Porcheron et al. (2013) review the enterobacterial metallo-transporters and their regulation, discussing strain-specific differences. Troxell and Hassan (2013) review Fe-dependent regulations of transcription by the Ferric Uptake Regulator to control iron metabolism, oxidative stress defense and virulence. Finally, Troxell and Yang (2013) present the metallo-regulation in the causative agent of Lyme disease (*Borrelia burgdorferi*) a bacterium that does not require iron for its metabolism.

### Conflict of interest statement

The authors declare that the research was conducted in the absence of any commercial or financial relationships that could be construed as a potential conflict of interest.
